# The PRIME trial protocol: evaluating the impact of an intervention implemented in public health centres on management of malaria and health outcomes of children using a cluster-randomised design in Tororo, Uganda

**DOI:** 10.1186/1748-5908-8-114

**Published:** 2013-09-30

**Authors:** Sarah G Staedke, Clare IR Chandler, Deborah DiLiberto, Catherine Maiteki-Sebuguzi, Florence Nankya, Emily Webb, Grant Dorsey, Moses R Kamya

**Affiliations:** 1 Department of Clinical Research, London School of Hygiene & Tropical Medicine, Keppel Street, London WC1E 7HT, United Kingdom; 2 Infectious Disease Research Collaboration, PO Box 7475, Kampala, Uganda; 3 Department of Global Health and Development, London School of Hygiene & Tropical Medicine, 15-17 Tavistock Place, London WC1H 95H, United Kingdom; 4 Department of Infectious Disease Epidemiology, London School of Hygiene & Tropical Medicine, Keppel Street, London WC1E 7HT, United Kingdom; 5 Department of Medicine, University of California, San Francisco, Box 0811, San Francisco, California, 94143, USA; 6 Department of Medicine, Makerere University, Kampala, Uganda

**Keywords:** Malaria, Complex intervention, Cluster-randomised trial, Uganda, Public sector

## Abstract

**Background:**

In Africa, inadequate health services contribute to the lack of progress on malaria control. Evidence of the impact of interventions to improve health services on population-level malaria indicators is needed. We are conducting a cluster-randomised trial to assess whether a complex intervention delivered at public health centres in Uganda improves health outcomes of children and treatment of malaria, as compared to the current standard of care.

**Methods/Design:**

Twenty public health centres (level II and III) in Tororo district will be included; 10 will be randomly assigned to the intervention and 10 to control. Clusters will include households located within 2 km of health centres. The trial statistician will generate the random allocation sequence and assign clusters. Health centres will be stratified by level, and restricted randomisation will be employed to ensure balance on cluster location and size. Allocation will not be blinded. The intervention includes training in health centre management, fever case management with use of rapid diagnostic tests (RDTs) for malaria, and patient-centered services, and provision of artemether-lumefantrine (AL) and RDTs when stocks run low. The impact of the intervention on population-level health indicators will be assessed through community surveys conducted at baseline in randomly selected children from each cluster, and repeated annually for two years. The impact on individuals over time will be assessed in a cohort study of children recruited from households randomly selected per cluster. The impact on health centres will be assessed using patient exit interviews, monthly surveillance, and assessment of health worker knowledge and skills. The primary outcome is the prevalence of anaemia (haemoglobin <11.0 g/dL) in individual children under five measured in the annual community surveys. The primary analysis will be based on the cluster-level results.

**Discussion:**

The PRIME trial findings will be supplemented by the PROCESS study, an evaluation of the process, context, and wider impact of the PRIME intervention which will be conducted alongside the main trial, together providing evidence of the health impact of a public sector intervention in Uganda.

**Trial registration and funding:**

This trial is registered at Clinicaltrials.gov (NCT01024426) and is supported by the ACT Consortium.

## Background

Malaria is a focus of Millennium Development Goal 4, aiming to reduce the mortality rate in children under five by two-thirds, between 1990 and 2015 [1]. In the past decade, increased donor financing and widespread scale-up of malaria control measures have substantially reduced the malaria burden in several countries [2-7]. However, these findings have not been consistent across Africa [8], and malaria-associated morbidity and mortality remains high in some countries, including Uganda [9-11]. In 2010, the World Health Organization (WHO) released new guidelines for malaria diagnosis and treatment recommending that suspected cases be confirmed by a parasitological test prior to treatment, when possible [12]. Although WHO’s call for universal diagnostic testing combined with increased availability of rapid diagnostic tests (RDTs) for malaria and artemisinin-based combination therapies (ACTs) should translate into improved malaria control, in many areas, health system challenges prevent achieving this in practice [1,13]. One of the main clinical challenges has been the management of negative RDT results, and the need to expand malaria training packages to include management of non-malaria febrile illnesses [14,15]. A more comprehensive approach to healthcare is called for to improve management of malaria and other febrile illnesses, and attract patients to seek care.

Systematic reviews of evaluations of training-based interventions aiming to change health worker behavior and improve quality of care have produced mixed results in both developed [16-19], and developing countries [20]. Despite the emphasis on training, clinical quality of care remains poor in many low-resource settings [21,22]. The failure of many training-based programs to improve clinical care reflects a wider acknowledgement of a gap between knowledge and practice of health workers. Further research into how to support clinicians to improve performance beyond didactic training is urgently needed.

In our formative research, we identified barriers and aspirations for quality healthcare in Tororo and evaluated these results with an aim to identify intervention options that could be feasibly implemented, and might have the greatest impact on health outcomes [22]. These findings were considered in the context of reviews of the literature on previous interventions and theory of behavior change and adult learning, and were discussed with stakeholders in Uganda. Using this information, we designed an intervention that could be sustainable by the Ministry of Health (MoH) and district partners in Uganda, which aims to attract patients to seek care and improve the quality of care delivered at public health centres. The intervention has four components including: training in-charges in health centre management; training to health workers in fever case management and use of RDTs; training health workers in patient-centered services; and ensuring adequate supplies of artemether-lumefantrine (AL) and RDTs. The manuals for delivering the intervention are available online at http://www.actconsortium.org/pages/resources-prime-trainer-and-learner-manuals-110.html. The intervention components are based on a theory that behavior change would occur as a cognitive, emotional, and social process, occurring in a community of practice [23,24], and supported by physical resources.

## Methods/Design

### Objectives and hypotheses

1. To compare the impact of the PRIME intervention with current standard of care on key population-based indicators, including the prevalence of anaemia in children under five (Table 1). We will test the primary hypothesis that the prevalence of anaemia will be lower in individual children under five from clusters randomised to the intervention than in children randomised to standard care. We will also test the secondary hypothesis that the prevalence of parasitaemia in children under five, and children aged 5 to 15 years, from clusters that are randomised to the intervention will be lower than in children randomised to standard care.

2. To compare the impact of the PRIME intervention with current standard of care on key longitudinal indicators, including treatment incidence density, in a prospectively followed cohort of children under five. We will test the hypothesis that delivery of antimalarial treatment via current care will result in over-treatment and a higher incidence of antimalarial treatment in individual children, than antimalarial treatment delivered from health centres randomised to the intervention, which should be targeted to test-confirmed cases of malaria.

3. To compare the impact of the PRIME intervention with current standard of care on key indicators of case management for malaria and other illnesses, including the risk of inappropriate antimalarial treatment, in children under five treated at health centres. We will test the hypothesis that the intervention decreases inappropriate treatment with ACTs, as measured by the proportion of individual children under five with suspected malaria and a negative RDT result that are inappropriately treated with an ACT plus the proportion of children under five with suspected malaria and a positive RDT result that are not prescribed an ACT at each health centre. We expect inappropriate treatment with ACTs to be lower in the health centres randomised to the intervention than in those in the standard care group.

**Table 1:** Study objectives and populations

**Objective**	**Study population and sample size**
1. To compare the impact of the PRIME intervention to current standard of care on key population-based indicators, including the prevalence of anaemia in children under five	Cross-sectional community surveys in children under five and aged 5 to 15 years randomly selected from households in each cluster (8,766 children total); surveys will be conducted at baseline and then annually for two years (three surveys in total)
2. To compare the impact of the PRIME intervention to current standard of care on key longitudinal indicators, including treatment incidence density, in a prospectively followed cohort of children under five	Cohort of children under five recruited from 25 households randomly selected from each cluster (500 total) and followed for approximately 18 months in total, 12 months following the implementation of the intervention; all children of appropriate age from each household will be eligible to participate
3. To compare the impact of the PRIME intervention to current standard of care on key indicators of case management for malaria and other illnesses, including the risk of inappropriate antimalarial treatment, in children under five treated at health centres	Exit interviews in patients attending public health centres (20 HC IIs and IIIs) in the study area (three surveys in total). In the first two surveys, including 10 patients per health facility (200 patients per survey). In the final survey, including 50 patients per health facility (1,000 patients in survey, 1,400 patients overall)

### Study site and population

**Figure 1:**
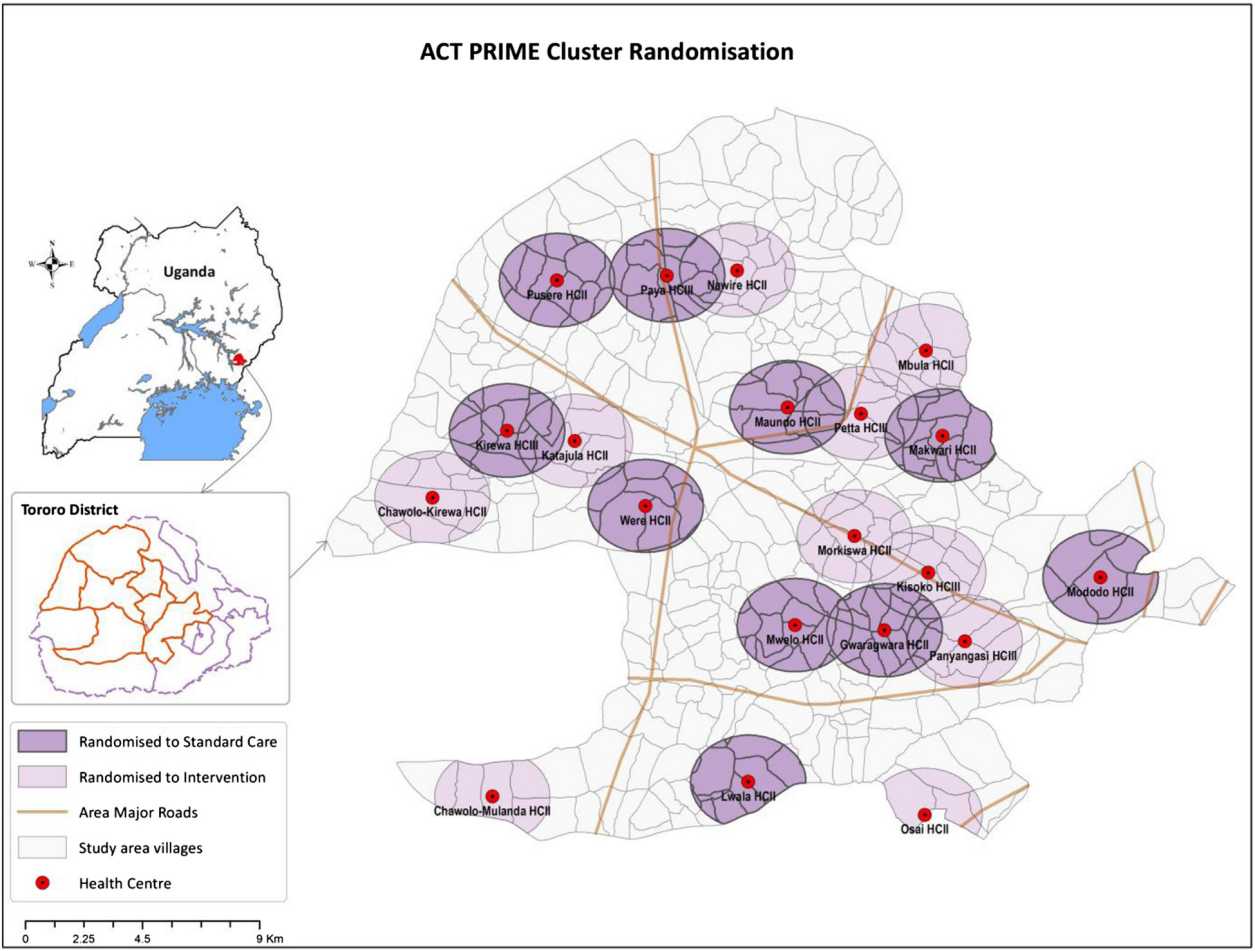
PRIME study area, health centres, and clusters.

Tororo district, eastern Uganda, is an area of high malaria transmission intensity (estimated entomologic inoculation rate of 562 infective bites per person-year) [25]. Seven sub-counties will be included (Figure 1). The study area is rural with limited infrastructure and education levels [26]. Very few households in the study area have electricity (1%). Health centres in the study area are generally run by nurses or nursing assistants, and most are understaffed. Infrastructure at the health centres is also limited; most lack electricity and running water. Prior to the trial, delivery of supplies, including AL, to health centres in the study area has typically been unpredictable.

### Cluster randomisation

Twenty public health centres and their surrounding clusters will be included in the trial; 10 clusters will be randomly assigned to the intervention and 10 to control. The cluster-randomised design was selected because the intervention will be implemented at health centres, while the primary outcome will be measured at the community level.

Government-run health centres (levels HC II and HC III) will be the unit of randomisation. Clusters will be defined prior to randomisation using a census database, and will include households located within 2 km of the health centres. Households will be excluded from our sampling frame if they are ≥ 2 km from any health centre. If a household is within 2 km of more than one health centre, the household will be assigned to the cluster of the closest health centre.

Of 22 health centres in the study area, two pairs of health centres have substantially overlapping catchment areas; one facility from each pair will be randomly excluded. Health centres will be stratified by level, and because of uneven numbers of HC IIs and IIIs, one of the HC IIIs without a laboratory will be 'demoted’ and paired with a HC II to ensure even numbers. Restricted randomisation will be employed to ensure balance on geographical location and cluster size. The trial statistician will generate the random allocation sequence using random number generation in R (http://www.r-project.org/), and will assign clusters to study arms. Study personnel will enroll clusters, and allocation will not be blinded.

### Sensitisation and recruitment of health centres

Key stakeholders, including national and district officials and community representatives, will be sensitised about the study design, selection of health centres, objectives, and procedures. Each health centre will be approached individually to discuss study participation, after randomisation. An information sheet will be used to describe the study and procedures, which will emphasise that study participation is voluntary and that health centres may withdraw at any time. Health centre in-charges will have an opportunity to ask questions and will be asked to provide verbal consent to participate on behalf of their health centre. Information about the trial will be provided at any time throughout the trial period, along with the opportunity to cease participation, in line with ethical practice of ongoing consenting procedures.

## PRIME intervention

### Overview

**Figure 2:**
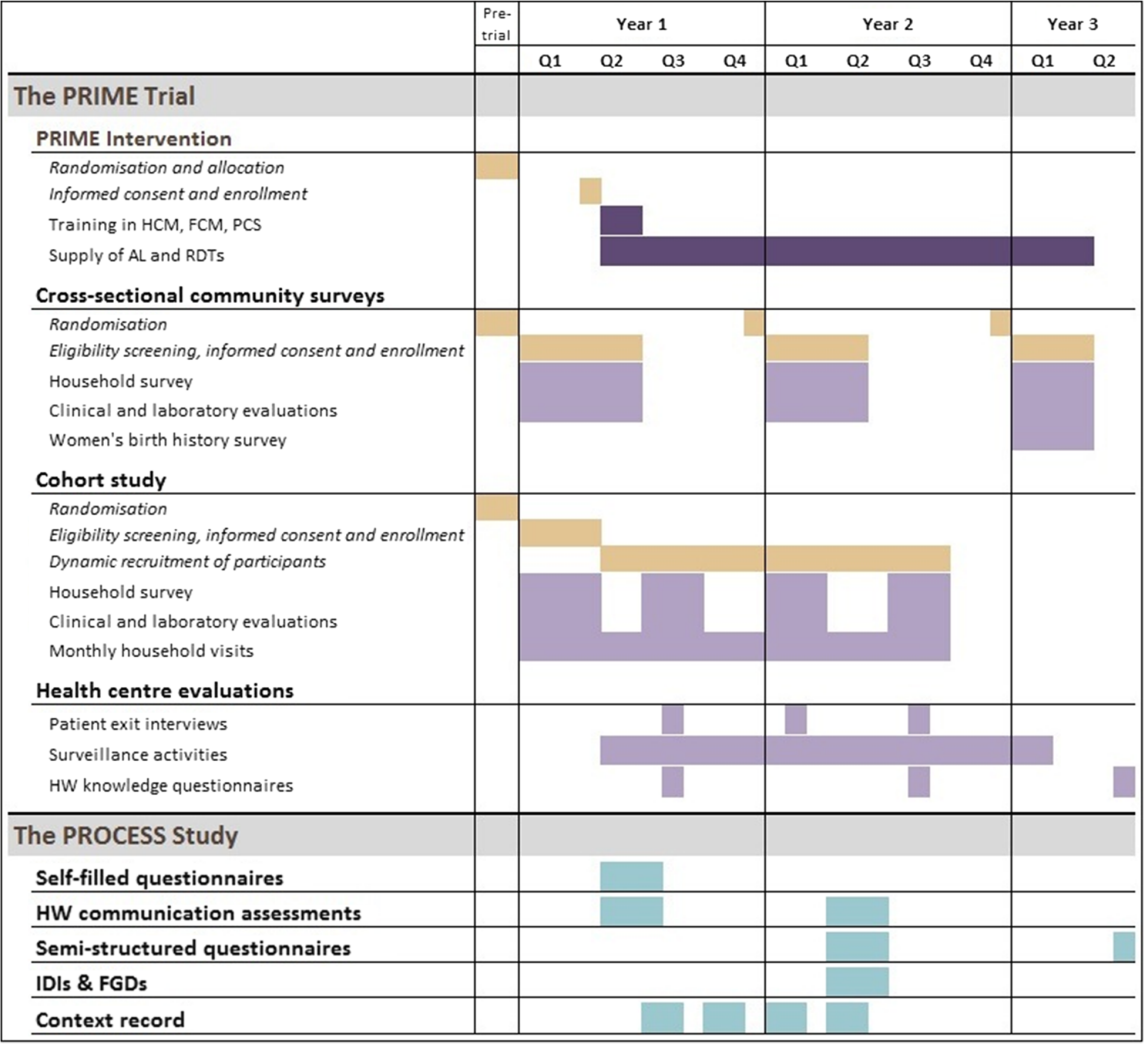
Trial timeline.

The intervention is designed to stimulate behavior change and build capacity through training of in-charges and health workers using adult learning techniques, and to ensure adequate supply of drugs and diagnostics at public health centres. Training sessions will be led by skilled trainers and health workers will be trained in two groups to ensure that clinical work at the health centres continues alongside the training. Training workshops will be held in convenient locations for the participants, and all costs will be covered and documented by the project. Training packages will be delivered over approximately 8 to 10 weeks; support for AL and RDTs will continue for the duration of the trial (Figure 2).

### Training in health centre management

Training in health centre management (HCM) aims to equip in-charges with key skills and tools required to effectively and efficiently manage their health centre. The HCM training package includes three workshops—financial management, supply management, and information management (http://www.actconsortium.org/pages/resources-prime-trainer-and-learner-manuals-110.html). The workshops will incorporate both didactic teaching and learner-centred activities including interactive practical sessions. Each workshop will include a half-day session once a week for three consecutive weeks.

### Training in fever case management

Training will be provided by the Joint Uganda Malaria Training Program (JUMP) team [27] using the RDT training guidelines [28], and RDT job aide, which have been adopted and implemented by Uganda’s MoH. The training includes sessions on evaluating febrile patients, performing and reading an RDT, management of patients based on RDT results, recognising and referring of patients with severe illness, RDT storage and monitoring, and infection prevention. Training will be conducted by the JUMP team over two weeks; the first two days will focus on theory, followed by on-site training at the health centres the next week. Additional on-site support supervision will be provided by the JUMP team six weeks and six months after the initial training, as part of the standard JUMP training.

### Training in patient-centred services

This training package aims to improve interpersonal interactions between health workers and patients by helping health workers to identify interpersonal challenges and develop skills for communicating and interacting with patients and colleagues. The Patient-Centered Services (PCS) training package includes six workshops: Introduction to PCS; Improving interactions with patients – Part 1; Improving interactions with patients – Part 2; Building a positive work environment; Improving the patient visit for health workers; and Improving the patient visit for support staff (http://www.actconsortium.org/pages/resources-prime-trainer-and-learner-manuals-110.html). The training includes self-observation activities to be carried out by health workers prior to each session, with an emphasis on building on individual and group experiences in learning through interactive workshops. Each workshop will include a half-day session led by a skilled trainer once a week for six consecutive weeks.

### Supply of AL and RDTs

The project will supplement the supply of AL and RDTs for malaria to ensure adequate stocks of these key commodities. Through Uganda’s National Medical Stores (NMS), health centres are supplied with standardised 'kits’ of medical supplies and drugs, every two months. If the provision of AL and RDTs by NMS is not adequate to meet demand at health centres, or if the NMS supply chain fails, the project will supply additional AL and RDTs. We will attempt to supply the same brand of RDTs provided by NMS (generally SD Bioline Pf, or SD Bioline Pf/PAN, Standard Diagnostics, Inc.).

A health sub-district liaison officer will be recruited to receive and process requisitions for AL and RDTs from health centres, aiming to mimic the current district procedures of direct delivery to health centres. The intervention will use the standard health management information systems (HMIS) supply management tools and procedures, which are included in the HCM training. Supply support via the liaison officer will begin as the training package is rolled out, and will continue until the final community survey is completed.

### Standard care

Standard care will include services typically provided by public health centres. No additional training will be provided to the in-charges or health workers stationed at these health centres; and no support for staffing or supplies will be provided beyond what is supplied by the district and MoH.

## Methods of evaluation

### Cross-sectional community surveys

At baseline and then annually for two years, community surveys will be conducted with 8,766 children, including 4,383 under-five and 4,383 aged 5 to 15 years, to assess the impact of the intervention on key population-level health indicators, including prevalence of anaemia (Table 2). New populations of children will be selected for each survey, which will include a structured questionnaire administered to the primary caregiver, and a clinical and laboratory assessment of each participating child.

**Table 2:** Primary and secondary outcomes

**Outcomes**	**Indicator**
**Objective 1: Cross-sectional community surveys**	
Prevalence of anaemia	Proportion of Hb measurements <11.0 g/dL. Anaemia will be classified according to severity: mild (Hb 8.0 – 10.9), moderate (Hb 5.0 – 7.9), severe (Hb <5.0).
Prevalence of parasitaemia	Proportion of thick blood smears that are positive for asexual parasites
Prevalence of gametocytaemia	Proportion of thick blood smears that are positive for gametocytes
All-cause mortality	Probability of dying between birth and five years of age, expressed per 1,000 live births
**Objective 2: Cohort study**	
Antimalarial treatment incidence density	Number of antimalarial treatments given for fever/malaria over the period of follow-up
Incidence of illness episodes	Episode of illness as reported by primary caregiver
Incidence of febrile episodes	Episode of illness associated with fever as reported by primary caregiver
Prompt effective treatment of fever	Proportion of children with fever treated within 24 hours of onset of symptoms with an ACT
Prompt effective treatment of malaria	Proportion of children with malaria (confirmed by a parasitological test) treated within 24 hours of onset of symptoms with an ACT
Incidence of serious adverse events	Any experience that results in death, life-threatening experience, hospitalisation, persistent or significant disability or incapacity, or specific medical or surgical intervention to prevent one of the other serious outcomes
Antibiotic treatment incidence density	Number of antibiotic treatments given for fever/bacterial illnesses over the period of follow-up
**Objective 3: Health facilities**	
**Patient exit interviews**	
Inappropriate treatment of malaria	Proportion of children under five with suspected malaria and a negative RDT result who are inappropriately given an ACT + Proportion of children under five with suspected malaria and a positive RDT result who are not prescribed an ACT
Appropriate treatment of malaria	Proportion of children under five with suspected malaria and a positive RDT result who are appropriately given an ACT + Proportion of children under five with suspected malaria and a negative RDT result who are not prescribed an ACT
Inappropriate treatment of malaria	Proportion of children under five with suspected malaria and a positive RDT result who are inappropriately given a non-ACT regimen
Patient satisfaction with healthcare	Proportion of patients indicating they were satisfied with care provided at the health centre in exit interviews
**Health facility surveillance**	
Patient attendance	Total number of patients attending health facilities and their characteristics, including age, gender, village of residence, and diagnosis
Gaps in staffing requirements	Required positions, as indicated by the MoH staffing norms policy, which are unfilled for greater than one month
Stock-outs of ACTs	Days per month that AL supplied by NMS via the district is not available
**Health worker knowledge questionnaire**	
Knowledge questionnaire scores	Proportion of questions answered correctly following training in fever case management

### Recruitment

A sampling frame for the surveys will be generated using the census database. All households enumerated during the census will be assigned a unique number. A random sample of households with at least one child under fifteen years of age will be selected from each cluster to generate a list of households to be approached. Three separate lists will be generated prior to each survey from the original census list.

Study personnel will conduct door-to-door recruitment. When a household with at least one child of appropriate age is identified, study personnel will describe the purpose of the study in the appropriate language (usually Japadhola, Luganda or Swahili) with parent(s) or guardian(s), and proceed with screening (Table 3). One child under five and one child aged 5 to 15 years will be eligible for participation from each household. If more than one child of appropriate age resides in the household, study personnel will record the gender and ages of all children under five and all children between the ages of 5 to 15 years, and one child from each age category will be randomly selected for participation using pre-defined guidelines.

**Table 3:** Selection criteria for community surveys, cohort study, and patient exit interviews

**Study component**	**Inclusion criteria**	**Exclusion criteria**
**Cross-sectional community survey**	1) age <15 years	1) inability to locate the child
	1) agreement of parents or guardians to provide informed consent	
	2) agreement of a child aged 8 years or older to provide assent	
**Cohort study**	1) age <5 years	1) intention to move during the follow-up period
	1) fagreement of parents or guardians to provide informed consent	1) current enrollment in another research study
**Patient exit interviews**	1) age <5 years	None
	2) agreement of parents or guardians to provide informed consent	

### Survey procedures

Primary caregivers will be surveyed to gather information about bednet use and management of fever within the last two weeks in any child under the primary caregiver’s care. In the final survey, all women of child-bearing age (13 to 49 years) in the household will be asked to provide birth histories, which will allow us to estimate all-cause mortality in children under five.

In each survey, participating children will undergo a brief history and physical examination, including measurement of temperature, weight, mid-upper arm circumference, and spleen size. Blood will be collected by fingerprick for thick blood smear and haemoglobin (all surveys), and for storage on filter paper for future molecular testing (baseline survey only).

### Sample size estimates

The primary outcome for community surveys, and the overall trial, is the prevalence of anaemia (haemoglobin <11.0 g/dL) in children under five (Table 2). Children under five will be sampled from each study cluster in proportion to the total cluster size, with a planned harmonic mean of 200 children per cluster. Using methods for a stratified, cluster-randomised design [29], and assuming a prevalence of anaemia of 65% at baseline [30], with a coefficient of variation (*k*) between clusters of 0.2, this sample size will allow us to detect an absolute difference in anaemia prevalence between study arms of 17% (or more) with 80% power at a 5% significance level. If we assume that the clusters are more homogeneous (*k* = 0.1), then a difference of 10% (or more) can be detected.

Prevalence of parasitaemia is a secondary outcome of the community surveys (Table 2). In children aged 5 to 15 years, the prevalence of parasitaemia is estimated to be 60% at baseline [30]. Based on this, and assuming *k* = 0.2, we estimate that a harmonic mean of 200 children aged 5 to 15 years surveyed in each cluster will allow us to detect an absolute difference in the prevalence of parasitaemia between study arms of 16%, at a 5% significance level with 80% power.

We had originally planned to recruit 200 children under five and 200 children aged 5 to 15 years from each cluster for the community surveys. However, upon completing the census, we discovered that the population of three clusters was smaller than expected. To adjust for the variability in population size between the clusters, we opted to weight the target sample size for the community survey according to the total population of each cluster to achieve a harmonic mean of 200 for each age category, resulting in different sample sizes for each cluster.

### Cohort study

A cohort of children under five will be enrolled from 25 households randomly selected from each cluster, for a total of 500 households, to assess the impact of the intervention on key longitudinal health indicators, including antimalarial treatment incidence density (Table 2). The cohort will be dynamic, and all children who are born, or move into, a participating household will be eligible for recruitment. Assessments will include clinical and laboratory evaluations, household surveys, and monthly household visits to gather information on management of illnesses. Participants will be followed for approximately 18 months in total, the equivalent of approximately 12 months following implementation of the intervention.

### Recruitment

A separate list of households to be approached for recruitment into the cohort study will be generated from the census database using the same methods as described for the community surveys. Study personnel will conduct door-to-door recruitment. When a household with at least one child of appropriate age is identified, study personnel will briefly describe the purpose of the study. If the parents (or guardians) are interested, a screening appointment will be scheduled. All children of appropriate age from a single household will be eligible for evaluation for study enrollment. The final clinical screening activities will be conducted at a study clinic. Children who meet the eligibility criteria (Table 3) will undergo a clinical and laboratory evaluation. Height, weight, and temperature will be measured, and spleen size will be evaluated. Blood will be collected by finger prick for thick blood smear, haemoglobin, and for storage on filter paper for future molecular testing.

### Study procedures

Clinical and laboratory evaluations of cohort participants will be repeated every six months. Following enrollment, or within two weeks period from enrollment, a household survey will be administered. Primary caregivers will be asked to complete a survey questionnaire (similar to that administered to in the community survey) to gather information about bednet use and management of febrile children. The household survey will then be repeated approximately 12 months after enrollment.

Primary caregivers will be asked to keep a diary of health of study participants for the duration of follow-up. The diaries will be based on instruments previously developed and validated in studies in Uganda and elsewhere in Africa [31,32]. The diaries have been developed by a Ugandan artist with input from the community, and will be used to collect information on clinical symptoms and healthcare expenditures. Households will be visited by study personnel every two weeks during the first two months, and then monthly, to collect completed diaries.

At each monthly visit, questionnaires will also be administered to gather additional data on the health of participants, management of any illnesses, and healthcare expenditures. The information collected in the diaries and the questionnaires will be complementary. Small incentives (including sugar, soap, or washing powder) will also be provided to each household during the monthly visit.

### Adverse event monitoring

Adverse events will be monitored in the cohort study. Data on serious adverse events (SAEs) and suspected adverse drug reactions will be collected retrospectively during the monthly household visits. Reports of SAEs that are classified as at least 'possibly’ related to administration of AL, and reports of all suspected unexpected serious adverse reactions (SUSARs) will be submitted to the institutional review boards (IRBs) according to their guidelines for expedited reporting. All serious adverse event reports and summary reports of suspected adverse drug reactions will be submitted to the IRBs, the Data and Safety Monitoring Board, and the ACT Consortium Drug Safety Register annually.

### Sample size estimates

The primary outcome for the cohort study will be treatment incidence density among children under five, which is assumed to be 2.5 treatments per year at baseline (Table 2). We conservatively estimate that at any one time the average number of children under five per household will be at least 1.6. Given this, 250 households, and at least 400 children at any one time, will be available in each intervention group. Since the cohort is dynamic, no allowance for losses to follow-up is required. Assuming that that treatment incidence remains constant in the standard care arm at 2.5 per year and *k =* 0.2, a total of 400 child-years of follow-up per arm (40 child-years per cluster) will allow us to detect a difference of one treatment per year between the two interventions at the 5% significance level with 80% power.

### Patient exit interviews

Exit interviews will be conducted with caregivers of children under five at all health centres to assess the impact of the intervention on malaria case management (Table 2). Three rounds of surveys are planned.

### Recruitment

Children and their caregivers will be approached by study personnel as they are leaving health centres. When a caregiver with a child of appropriate age is identified, study personnel will briefly describe the purpose of the study, and review the selection criteria (Table 3). All children of the same caregiver will be eligible to participate if of the appropriate age and seen by a health worker at the health centre.

### Study procedures

Caregivers will be interviewed using a standardised questionnaire to gather information about the purpose of the visit, presenting complaint, the child’s symptoms, whether a RDT or blood smear was done, the diagnosis given, medications prescribed, and medications received. Additional information about the satisfaction of the caregiver with the visit to the facility will also be obtained.

A clinical evaluation of the child will also be performed by a study physician as part of the exit interview. If the child has a temperature of ≥38.0°C or a history of fever in the past 48 hours, a fingerprick blood sample will be obtained to perform a RDT. Febrile children will be treated with paracetamol as appropriate. The results of the RDTs performed by the study personnel will be compared to the results of RDTs performed by health facility staff, where possible. Children with a positive RDT and no evidence of severe malaria, who have not been prescribed or received an ACT, will be given AL. Children with a positive RDT and evidence of danger signs of severe disease will be referred to an appropriate facility for further evaluation and treatment, regardless of the medicine prescribed.

### Sample size estimates

In the first two surveys, 10 patients will be selected by convenience sampling from each facility to participate in the interviews (200 total per survey). In the final survey, 50 patients will be recruited to participate (1,000 total in survey). In total, 1,400 patients will participate in the interviews.

The primary outcome for the patient exit interviews is inappropriate treatment of malaria at health centres in children under five, based on the result of a RDT performed by the PRIME team (Table 2). The hypothesis is that the proportion of children inappropriately treated with an ACT will be lower in intervention health centres compared to those in the standard care. In the original sample size calculations (for rounds one and two), we assumed this proportion to be 50% in the standard care group. Thus, interviewing 10 children and their caregivers in each of the 20 clusters would give 80% power to detect an absolute difference in the proportion inappropriately treated for malaria between the two intervention arms of 24% (or more) at the 5% significance level, assuming a coefficient of variation between clusters of 0.2 and allowing for the stratified design.

Although the original sample size calculations were based on the assumption that inappropriate treatment for malaria in the standard care group would be 50%, preliminary estimates suggest that only 35% of children in the standard care health centres have been inappropriately treated. Thus, for the final round of patient exit interviews, the sample size calculations have been revised. Assuming the proportion of children inappropriately treated to be 35% in the standard care arm with *k* = 0.2, interviewing 50 children and their caregivers in each of the 20 clusters will give 80% power to detect an absolute difference of 12% (or more) at the 5% significance level.

### Health centre surveillance

Surveillance activities will be conducted at all health centres initially every month, to collect information about patient attendance, drug stocks, staffing, and health centre costs from all centres. After the first year, data will be collected every two to three months. Data will be collected using a modified version of the HMIS outpatient department (OPD) register, developed in collaboration with the MoH, and the drug stock card. In addition, the in-charge will be interviewed to gather information on factors affecting health centre operations.

### Health worker knowledge questionnaires and skills assessment

Health workers at all participating health centres will be assessed about their knowledge of fever case management soon after the implementation of the intervention. The knowledge questionnaire includes structured questions allowing for open-ended answers to assess the health workers’ knowledge about malaria transmission, symptoms, diagnosis, and treatment, and etiology of non-malaria fevers. Additional assessment of health worker knowledge will be carried out using the pre- and post-training questionnaires administered by the JUMP team in their training on fever case management at approximately one year after the implementation of the intervention. Approximately two years after implementation of the intervention, assessment of health worker knowledge, and skills for performing RDTs will be conducted. Additional information about prior training on use of RDTs will be gathered, and health workers will be observed performing an RDT and managing a child with suspected malaria.

### Management of ill children

In the community surveys, cohort study, and patient exit interviews, children who are reported to have been febrile in the past 48 hours, or who have a temperature of ≥38.0°C, will have an RDT performed (SD Bioline Pf/PAN, Standard Diagnostics, Inc.). Febrile children will be treated with paracetamol as appropriate. Children with a positive RDT and no evidence of severe malaria will be treated with AL, if not already on appropriate treatment. Children with a positive RDT and evidence of danger signs of severe disease will be referred for further evaluation and treatment. Any child with haemoglobin <5.0 g/dL, or other concerning clinical symptoms will be referred for further management as appropriate.

## Laboratory procedures

### Microscopy

Thick blood smears will be stained with 2% Giemsa for 30 minutes and read by experienced laboratory technologists who are not involved in direct participant care. For the cohort study and first community survey, parasite densities will be calculated by counting the number of asexual parasites per 200 leukocytes (or per 500 leukocytes, if the count is <10 asexual parasites/200 leukocytes), assuming a leukocyte count of 8,000/μl. A blood smear will be considered negative when the examination of 100 high power fields does not reveal asexual parasites. For the second and final community survey, thick blood smears will be read only for presence or absence of asexual parasites, parasite density will not be determined. Gametocytaemia will also be determined from thick smears. For quality control, all slides will be read by a second microscopist and a third reviewer will settle any discrepant readings.

### Haemoglobin measurement

Haemoglobin will be measured from fingerprick blood samples using a portable spectrophotometer (HemoCue, Anglom, Sweden).

### Molecular studies

A filter paper sample will be collected each time a thick blood smear is obtained in the baseline cross-sectional survey and cohort study. Blood will be placed onto filter paper in approximately 25 μl aliquots per blood spot (four blood spots per sample). The samples will be labeled, air-dried, and stored at ambient temperature with desiccant. Parasite DNA will subsequently be removed from the filter paper and prepared for molecular analysis using a chelex extraction method. Molecular studies may include analyses of polymorphisms in parasite and/or human genes for mutations that may impact on clinical malaria, and genotyping of malaria parasites, and will have no impact on the clinical management of study participants.

### Analytical plan

Analysis will be conducted at both the cluster level using summary statistics from each cluster, and at the individual level. The primary analysis will be based on the cluster-level results as this is expected to be more robust when the number of clusters randomised is not large. A two-stage approach based on cluster summaries will be used to adjust for individual- and cluster-level covariates, where appropriate.

### Cross-sectional community surveys

Data from each community survey will be analysed separately. The crude prevalence of anaemia will be tabulated for each cluster. A weighted average of the cluster prevalences will also be calculated for comparison, with the weights provided by the sample size for each cluster. A risk ratio for the effect of the intervention will be calculated directly from the cluster-based point estimates. If necessary, a logarithmic transformation will be applied to normalise cluster-specific prevalences before analyzing the data. A stratified t-test will be used to compare the means of the cluster-specific proportions, where the within-stratum between-cluster variance will be estimated as the residual mean square from a two-way analysis of variance of the log-prevalences on stratum and treatment arm, including an interaction term. A 95% confidence interval (CI) for the risk ratio, adjusting for stratum, will be calculated from this variance using a t-statistic with 16 degrees of freedom.

Adjustment for baseline imbalances between groups, cluster-specific prevalence of anaemia collected at the baseline community survey, and *a priori* individual-level factors (age, gender, use of ITNs, and distance to the health centre), will be conducted using a two-stage process. At the first stage, a logistic regression model, including terms for stratum and the covariates to be adjusted for, but excluding the intervention effect, will be fitted to calculate cluster-specific predicted prevalences. The ratio between the observed and predicted prevalence will be calculated (risk ratio-residuals). At the second stage, methods described above for estimating the 95% CI and performing a stratified t-test will be conducted with the cluster-level prevalences replaced with the covariate-adjusted ratio-residuals.

### Cohort study

The number of events, child-years of follow-up, and corresponding treatment incidence rate will be tabulated by cluster. For each study arm, the cluster-specific rates will then be averaged to give a point estimate of the rate for each intervention. Rate ratios for the effect of the intervention on each outcome will then be calculated from these point estimates. The distribution of cluster-specific rates is likely to be skewed, therefore a logarithmic transformation will be applied to normalise rates before analysis. Methods for estimating the 95% CI and a formal test of the null hypothesis that the rate ratio is equal to one will be conducted. Adjusted analysis will be conducted using the two-stage process described above, except Poisson regression will be used to calculate predicted rates and rate ratio residuals. *A priori* individual-level factors to be adjusted for are baseline anaemia, age, gender, use of ITNs, and distance to the health centre.

### Patient exit interviews

For each time point, the proportion of inappropriate treatment of malaria will be tabulated by cluster, and the cluster-specific mean proportions will be averaged to give a point estimate of proportion of participants appropriately treated with an ACT in each study arm. The risk ratio for the impact of the intervention will then be calculated. The two-way analysis of variance and stratified t-test approach described above for the community survey prevalence outcomes will then be applied to test the null hypothesis that the risk ratio equals one and to derive a 95% CI for the risk ratio. Adjusted analyses will also be performed to account for any baseline differences between the study arms, using the same two-stage approach described above. *A priori* individual-level factors to be adjusted for are age and gender.

### Data management

A combination of approaches will be used to collect data in this study, including use of paper forms, hand-held personal data assistants (PDAs), and tablet computers. Paper versions will be used for screening forms; clinical record forms for the baseline community survey and cohort study; and interview questionnaires for the first two rounds of patient exit interviews. Data entered onto paper record forms will be double-entered into a computerised database (Microsoft Access) to verify accuracy.

Data will be collected using questionnaires on PDAs (Visual CE, Syware Inc) in the baseline community survey (survey questionnaire), the cohort study (first household survey, monthly questionnaires), and health centre surveillance. Generally, field teams will move in pairs; one team member will administer the questionnaire and record answers on a PDA, while another will record answers on a paper questionnaire. Data captured on PDAs will be downloaded daily to a Microsoft Access database. Data captured on paper record forms will be used as back-up if synchronisation of the PDA to the computerised database fails.

Tablet computers will be used to collect data for the repeat community surveys, the second cohort household survey, the final round of patient exit interviews, and the final six months of health centre surveillance. Information from the questionnaires and fields for entering results of biomarker testing will be programmed into the tablets. Programming will include range, structure, and internal consistency checks. Data from these devices will be transferred at the end of every day to our core facility and stored on a secure server. Microscopy results will be recorded in a laboratory record book by lab technicians and will be double-entered into a computerised database (Microsoft Access) to verify accuracy.

### Ethical approval

The trial has been approved by the Ugandan National Council for Science and Technology (UNCST Ref HS 794), the Makerere University School of Medicine Research & Ethics Committee (SOMREC Ref 2010–108), The London School of Hygiene and Tropical Medicine Ethics Committee (LSHTM Ref 5779), and the University of California San Francisco Committee on Human Research (UCSF CHR Ref 006160). Sponsorship and insurance is provided by the LSHTM’s Clinical Trials Sub-Committee (Ref QA292).

### Informed consent

Study personnel will conduct informed consent discussions with all potential participants emphasising that the participation in the study is voluntary and that participants have the opportunity to withdraw from the study at any time without consequence. All information sheets and consent forms will be available in English, Japadhola, Luganda, and Swahili, and will be verified for accuracy through back-translation. Consent discussions will be conducted in the appropriate language and a translator will be used if necessary.

Verbal consent will be sought from health centre in-charges to participate in study, and to conduct evaluation activities (patient exit interviews, surveillance, and health worker knowledge and skills assessments) at their health centre. For the baseline community survey and the cohort study, written consent will be sought from parents (or guardians) for their child to participate in a research study and for the future use of biological specimens (filter paper samples). For the repeat community surveys and the patient exit interviews, written consent will be sought for study participation only, because no filter paper samples will be collected. If the parent or guardian is unable to read or write, their fingerprint will substitute for a signature, and a signature from a witness to the informed consent procedures will be obtained. Written assent to participate in the community surveys will also be obtained from children aged 8 years and older at the time of screening.

### Trial status

The PRIME trial field work completed in July 2013. Data cleaning and analysis of the final community survey, including the primary outcome for the survey and the overall trial, has not yet begun.

## Discussion

The PRIME trial will provide evidence on the impact and sustainability of an intervention to improve quality of healthcare, as compared to the current standard of care currently provided at public health centres, focusing on health outcomes in children and appropriate treatment of malaria. The PROCESS study, a mixed-methods evaluation, is also being conducted alongside the main trial [33]. The PROCESS study was designed to further our understanding about why the PRIME intervention is effective, or not. The PROCESS study includes an evaluation of the implementation of the intervention activities; mechanisms of change from the perspective of implementers, health workers, community members, and key stakeholders; a context evaluation to capture information on factors that may affect the implementation of the intervention or outcomes; and a limited impact evaluation to assess the wider impact of the intervention beyond outcomes of the PRIME trial. The results of the PRIME trial will be interpreted alongside the findings of the PROCESS study.

## Abbreviations

ACT: Artemisinin-based combination therapy; AL: Artemether-lumefantrine; HC: Health centre; HCM: Health centre management; HMIS: Health management information systems; JUMP: Joint Uganda malaria program; IRB: Institutional review board; LSHTM: London school of hygiene and tropical medicine; MoH: Ministry of health; MU: Makerere university (Kampala, Uganda); M&E: Monitoring and evaluation; NMS: National medical stores; OPD: Out-patient department; PCS: Patient-centered services; PDA: Personal data assistant; RDT: Rapid diagnostic test; SAE: Serious adverse event; SOMREC: School of medicine research and ethical committee (Makerere University); SUSAR: Suspected unexpected serious adverse reaction; UCSF: University of California, San Francisco; UNCST: Uganda national council of science and technology; WHO: World Health Organization.

## Competing interests

The authors declare that they have no competing interests.

## Authors’ contributions

SGS, CIRC, GD, and MK conceived of the study. CIRC, DD and SGS developed the intervention, with support from CMS and FN. SGS, DD, CMS, and FN drafted the protocol with CIRC, and with statistical support from EW. All authors reviewed the protocol and gave permission for publication.
